# Effect of testosterone replacement or duration of castration on baroreflex bradycardia in conscious rats

**DOI:** 10.1186/1471-2210-5-9

**Published:** 2005-03-30

**Authors:** Gregg R Ward, Abdel A Abdel-Rahman

**Affiliations:** 1 Department of Pharmacology, The Brody School of Medicine at East Carolina University, Greenville, NC, 27858, USA

## Abstract

**Background:**

In this study, we tested the hypothesis that 17β-estradiol contributes to testosterone-mediated restoration of baroreflex-mediated bradycardia in short-term (3 weeks) castrated rats. Further, a reported increase in serum testosterone after long-term (6 weeks) castration constituted a basis for testing the hypothesis that a spontaneous increase in serum testosterone or androstenedione in this model causes a commensurate increase in baroreflex-mediated bradycardia.

**Results:**

Testosterone (1 week) replacement enhanced baroreflex-mediated bradycardia in short-term castrated rats without changing 17β-estradiol level. A spontaneous recovery of baroreflex-mediated bradycardia occurred following long-term castration, although circulating testosterone and androstenedione remained suppressed.

**Conclusion:**

The data suggest: 1) 17β-Estradiol does not contribute to testosterone restoration of the baroreflex-mediated bradycardia in short-term castrated rats. 2) The long-term modulation of baroreflex-mediated bradycardia occurs independent of androgens, or the baroreflex mechanism may become adapted to low levels of circulating androgens.

## Background

Short-term castration attenuates the vagal component of baroreflex sensitivity in sexually mature male rats [1,2]. Factors influencing baroreflex sensitivity include gender, age, baseline mean arterial pressure and heart rate, anesthesia, and the method of drug administration [3-9]. However, these factors cannot account for the difference in baroreflex sensitivity between intact and castrated rats in reported studies [1,2].

El-Mas et al. [1,2] have shown that testosterone replacement restores baroreflex-mediated bradycardia in short-term castrated rats. Further, we and others [4,10] have demonstrated that 17β-estradiol enhances baroreflex sensitivity. Notably, 17β-estradiol is a product of testosterone aromatization by the aromatase enzyme [11], which raises its possible contribution to testosterone effects on baroreflex sensitivity. This latter possibility has not been investigated. Further, Ando et al. [12] demonstrated a reflex increase in circulating testosterone of adrenocortical origin subsequent to long-term (6 weeks) castration. The impact of such an increase in serum testosterone on baroreflex sensitivity is not known. Hence, the main objective of this study was to test the hypotheses that (i) 17β-estradiol, contributes to testosterone restoration of the vagal component of baroreflex sensitivity in short-term (3 weeks) castrated rats, and (ii) the spontaneous increase in serum testosterone, or androstenedione, reported by others [12] in long-term (6 weeks) castrated rats results in proportionate increase in baroreflex sensitivity. This goal was achieved by determining whether a) Testosterone replacement (1 week) restores the attenuated baroreflex sensitivity in short-term castrated rats, b) Serum 17β-estradiol increases in testosterone-treated rats, and c) An increase in baroreflex sensitivity occurs in long-term castrated rats along with an increase in serum testosterone or androstenedione. These studies were undertaken in conscious unrestrained rats to avoid the confounding effects of anesthetics on the measured variables [3].

## Results

### Effect of testosterone replacement on baroreceptor reflex control of heart rate

**Figure 1:**
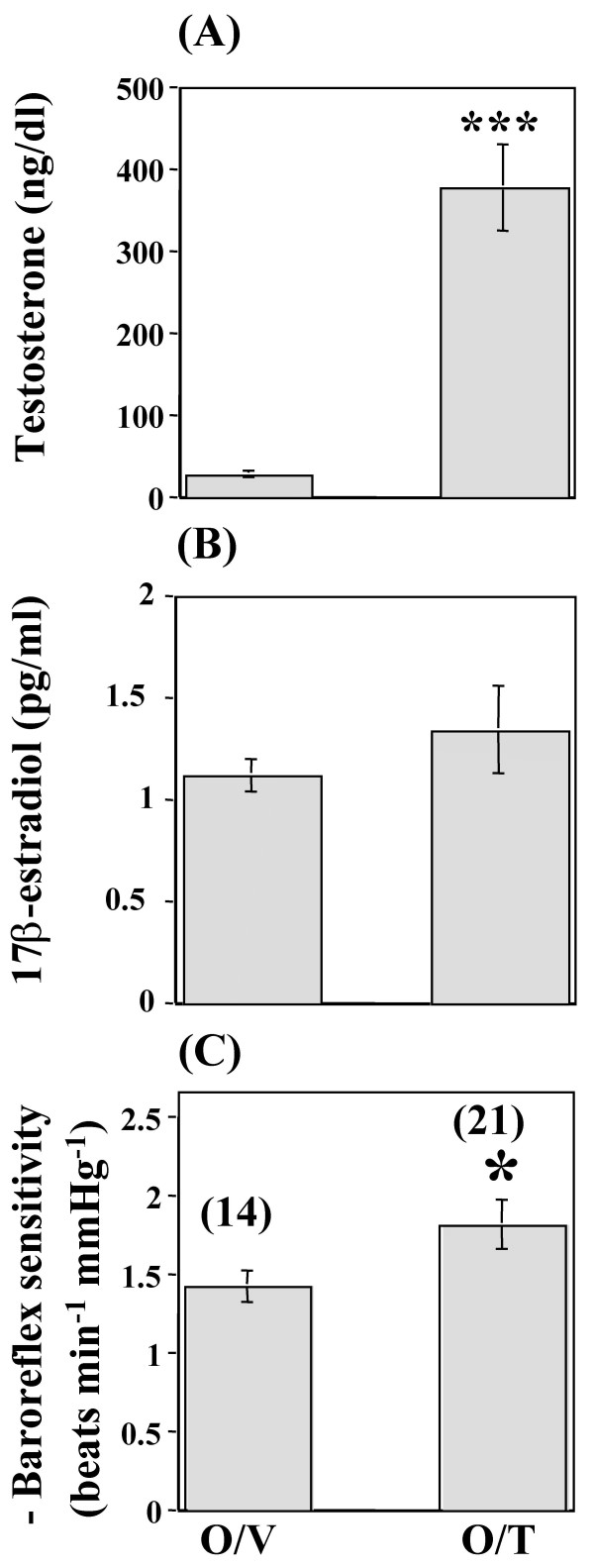
**Effect of testosterone depletion and replacement on baroreflex sensitivity and serum hormone levels**. Serum testosterone (A) and 17β-estradiol (B) level and baroreflex sensitivity (C) in conscious unrestrained male rats following orchiectomy (3 weeks) and 1-week treatment with testosterone or vehicle. Data are means ± SEM. Numbers in parentheses are number of observations. Orchiectomized/testosterone (O/T), orchiectomized/vehicle (O/V). * P < 0.05, *** P < 0.001 vs. orchiectomized/vehicle.

Baseline mean arterial pressure values, measured on the day of the experiment, were similar between the orchiectomized/testosterone and orchiectomized/vehicle rats (Table 1). However, the orchiectomized/testosterone rats exhibited a significantly (P < 0.05) lower baseline heart rate compared with the orchiectomized/vehicle rats (Table 1). Phenylephrine elicited similar rises in mean arterial pressure in all groups (data not shown). However, the similar increments in mean arterial pressure were accompanied by greater reflex bradycardic responses in the orchiectomized/testosterone compared with the orchiectomized/vehicle rats (data not shown). Therefore, baroreflex-mediated bradycardia was significantly enhanced in orchiectomized/testosterone compared with the orchiectomized/vehicle rats (-1.82 ± 0.15 vs. -1.43 ± 0.1 beats min^-1 ^mmHg^-1^; P < 0.05, Figure 1C). In addition, serum testosterone increased to within physiological levels in orchiectomized/testosterone rats (P < 0.001, Figure 1A). There was no change in serum 17β-estradiol in orchiectomized/testosterone compared with orchiectomized/vehicle rats (Figure 1B).

**Table 1:** Baseline mean arterial pressure (MAP) and heart rate (HR) of orchiectomized/testosterone (O/T), orchiectomized/vehicle (O/V), long-term castrated (L.T.C.), and sham-operated (SO) rats. The rats were allowed to acclimatize to laboratory conditions for at least 2 h prior to experimentation and a period of at least 30 min was allowed after connecting the rat to the pressure transducer for stabilization of blood pressure and heart rate. ^*a *^P < 0.05 vs orchiectomized/vehicle.

**Group**	**n**	**MAP (mmHg)**	**HR (beats/min)**
O/T	21	108 ± 2.6	392 ± 6^*a*^
O/V	14	105 ± 1.7	427 ± 10
L.T.C.	12	109 ± 4.1	414 ± 9
S.O.	5	102 ± 1.8	421 ± 14

### Effect of long-term castration on baroreceptor reflex control of heart rate

**Figure 2:**
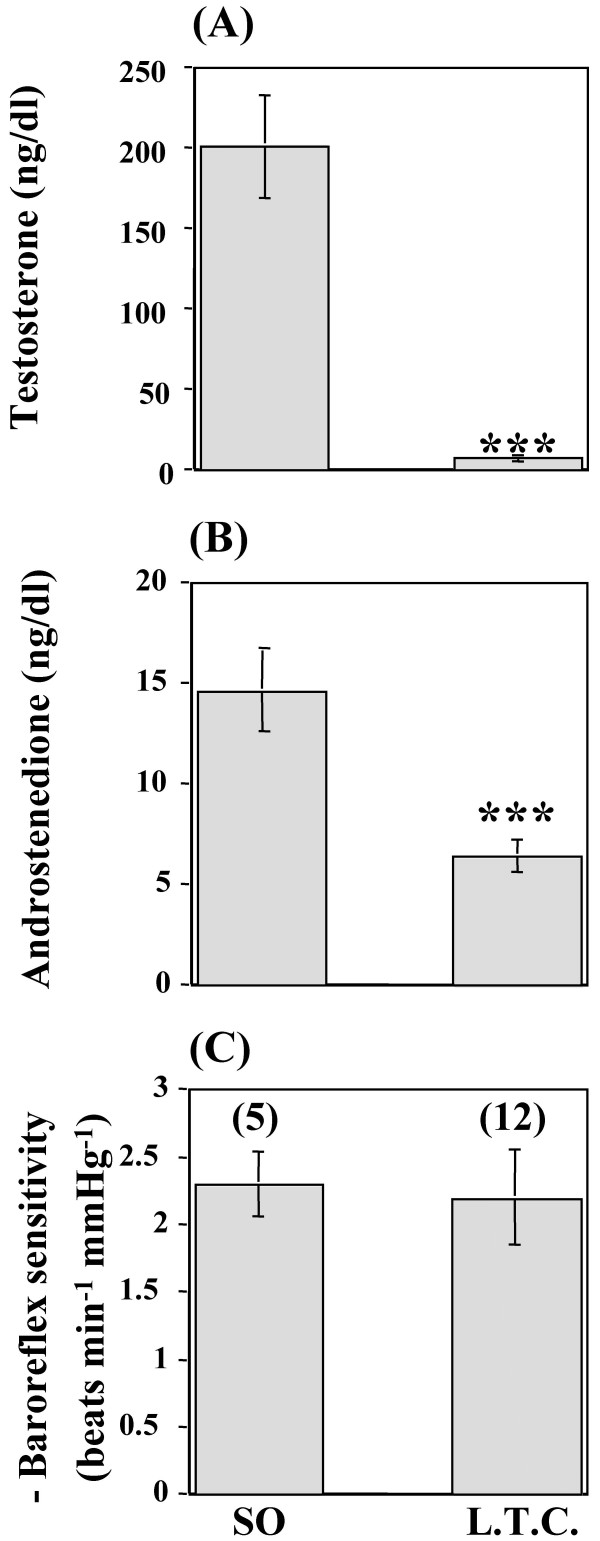
**Effect of long-term castration on baroreflex sensitivity and serum hormone levels. **Serum testosterone (A) and androstenedione (B) level and baroreflex sensitivity (C) in conscious unrestrained rats 6 weeks after castration (long-term castration or L.T.C.) or sham-operation (SO). Data are means ± SEM. Numbers in parentheses are number of observations. *** P < 0.001 vs. sham-operated.

Baseline mean arterial pressure and heart rate values, measured on the day of the experiment, were similar between the sham-operated and long-term castrated groups of rats (Table 1). Phenylephrine elicited similar rises in mean arterial pressure in all groups (data not shown). In addition, at any given rise in blood pressure, the reflex bradycardic response was similar in long-term castrated rats to that of the sham-operated rats (data not shown). Therefore, baroreflex-mediated bradycardia was restored compared with the sham-operated rats (-2.20 ± 0.35 vs. -2.29 ± 0.24 beats min^-1 ^mmHg^-1^; P > 0.05, Figure 2C). Finally, the restoration of baroreflex sensitivity subsequent to long-term castration occurred in the presence of significantly (P < 0.001) lower circulating levels of testosterone and androstenedione compared with the sham-operated rats (Figures 2A and 2B). Comparison of the data obtained from short-term vs. long-term castrated rats revealed that the baroreflex-mediated bradycardia underwent a phase of inhibition at the 3^rd ^week, but such inhibition was no longer evident by the 6^th ^week after castration (Figure 2C). Notably, serum testosterone depletion, observed 3 weeks after castration, continued to be evident 6 weeks after castration (Figure 2A). Therefore, no correlation was observed between baroreflex sensitivity and serum testosterone (r = 0.2, P > 0.05).

## Discussion

The current study presents 3 new findings. First, testosterone replacement (1 week) restores baroreflex sensitivity (baroreflex-mediated bradycardia) to control level, but 17β-estradiol does not contribute to this action in short-term (3 weeks) castrated rats. Second, baroreflex-mediated bradycardia spontaneously recovers to sham-operation level subsequent to long-term (6 weeks) castration, which suggests that the restoration of baroreflex-mediated bradycardia is time-dependent. Third, the spontaneous recovery of baroreflex sensitivity occurs in spite of significantly suppressed circulating levels of testosterone and androstenedione. This suggests that the long-term modulation of baroreflex sensitivity occurs independent of androgens, or the baroreflex mechanism may become adapted to low levels of circulating androgen.

In the present study we investigated the possibility that 17β-estradiol derived by the aromatization of testosterone [11] contributed to the testosterone-mediated increase in baroreflex-mediated bradycardia recently reported by El-Mas et al. [1,2]. Because Saleh and Connell [10] have shown that 17β-estradiol enhances the baroreflex-mediated bradycardia of male rats, we reasoned that testosterone enhancement of baroreflex sensitivity in castrated rats, might be secondary to the conversion of testosterone to 17β-estradiol. As previously shown [1,2] we demonstrated that testosterone replacement, which resulted in physiological levels of plasma testosterone, increased baroreflex-mediated bradycardia. Results of the present study showed that serum 17β-estradiol level did not change in testosterone-treated rats, which suggests that 17β-estradiol does not contribute to the action of testosterone on baroreflex control of heart rate following testosterone replacement. Hence, testosterone or another androgenic metabolite(s) seems to mediate the enhanced baroreflex response in short-term castrated rats. This notion is consistent with our preliminary finding that demonstrates the importance of the androgen receptor in the enhancement of baroreflex sensitivity in male rats [16].

The enhanced baroreflex-mediated bradycardia by testosterone replacement observed in this study and by others [1,2] together with the compensatory reflex increase in serum testosterone by the adrenal cortex, subsequent to long-term (6 weeks) castration [12], raised the interesting possibility that such an increase in endogenous testosterone may result in a proportionate increase in baroreflex-mediated bradycardia. Indeed, baroreflex-mediated bradycardia spontaneously recovered after long-term (6 weeks) castration to the sham-operation level. This finding supported our hypothesis and suggests that the restoration of baroreflex sensitivity after castration is time-dependent.

Contrary to our expectation and in disagreement with reported findings in a similar animal model [12], serum testosterone remained significantly suppressed in our animals. The discrepancy between both studies may be attributed to the antibody used by Ando et al. [12] to measure serum testosterone, which exhibited 40% cross-reactivity with dihydrotestosterone (a major product of testosterone) vs. 4% in our study. Nonetheless, the spontaneous and complete recovery of baroreflex-mediated bradycardia in the presence of significantly suppressed serum testosterone raised the following possibilities. Androgens other than testosterone (particularly androstenedione) may have enhanced baroreflex-mediated bradycardia subsequent to long-term castration (6 weeks), because (i) serum androstenedione increased to pre-castration levels 6 weeks after castration [12], and (ii) Labrie et al. [16] demonstrated that flutamide, a competitive nonsteroidal antiandrogen, which attenuated baroreflex-mediated bradycardia in our preliminary study [17], antagonized the effect of androstenedione at the androgen receptor. Since we observed no change in serum androstenedione subsequent to long-term castration, which again disagrees with reported findings [12], the present findings suggest that androstenedione, like testosterone, may not play a role in the spontaneous recovery of baroreflex-mediated bradycardia. The discrepancy between both studies may be attributed to a lower recovery rate (<65%) for androstenedione by Ando et al. [12] vs. a higher recovery rate (>80%) in this study.

An alternate explanation for the spontaneous recovery of baroreflex sensitivity in the presence of suppressed testosterone and androstenedione is that the baroreceptor reflex mechanism may have become adapted to the low level of circulating androgens. This would suggest an association between long-term castration, and the activation of compensatory mechanisms possibly involving an increase in androgen receptor density and/or signaling. Yu and McGinnis [18] reported a decrease in androgen receptor density in the nucleus ambiguus (one of the nuclei implicated in the baroreceptor heart rate response) [19] at 2 weeks castration, a period that coincides with a significant attenuation of baroreflex sensitivity observed in the present study and by others [1,2]. Whether an upregulation of androgen receptors in the brainstem occurs 6 weeks after castration remains to be investigated.

## Conclusion

Testosterone contributes to the maintenance of baroreflex-mediated bradycardia in adult rats. Such a physiological role for testosterone seems to manifest only on a short-term basis because: (i) Short-term (3 weeks) castration caused a significant reduction in baroreflex-mediated bradycardia, a deficit corrected by testosterone replacement. (ii) Spontaneous recovery of baroreflex-mediated bradycardia occurred following long-term (6 weeks) castration in spite of the continued suppressed testosterone and androstenedione levels, which may argue against a long-term effect of testosterone or androstenedione on baroreflex-mediated bradycardia. Circulating androgens other than testosterone and androstenedione may contribute to the restoration of baroreflex-mediated bradycardia subsequent to long-term castration. An alternate possibility, that remains to be investigated, is that the baroreceptor reflex mechanism may have become adapted to the low level of circulating androgens.

## Methods

### Preparation of the rats

Male Sprague-Dawley rats (Harlan Farms, Indianapolis, IN) were used in this study. Arterial blood pressure was measured according to the method used in our previous studies [4]. Briefly, the rats were anesthetized with methohexital sodium (50 mg kg^-1^, i.p.). Catheters (polyethylene 10 connected to polyethylene 50), which were filled with heparinized saline (100 U ml^-1^), were placed in the abdominal aorta and vena cava via the femoral artery and vein for measurement of blood pressure and i.v. administration of drugs, respectively. The catheters were inserted about 5 cm into the femoral vessels and secured in place with sutures. Finally, the catheters were tunnelled s.c., exteriorized at the back of the neck between the scapulae, and plugged by stainless steel pins. Incisions were closed by surgical staples and swabbed with povidone-iodine solution. Each rat received s.c. injection of buprenorphine hydrochloride (Buprenex; 0.3 μg rat^-1^) to control pain and an i.p. injection of 50,000 U kg^-1 ^of penicillin G benzathine and penicillin G procaine in an aqueous suspension (Durapen) and was housed in a separate cage. The experiment was started 48 h later, which involved the connection of the arterial catheter to a Gould-Statham pressure transducer (Oxnard, CA). The blood pressure was displayed on a Grass polygraph (model 7D, Grass Instruments Co., Quincy, MA). Heart rate was computed from blood pressure waveforms by a Grass tachograph and was displayed on another channel of the polygraph.

Experiments were performed in strict accordance with institutional animal care and use guidelines, and in accordance with the principles and guidelines of the National Institutes of Health Guide for the Care and Use of Laboratory Animals.

### Orchiectomy

Bilateral orchiectomy was performed as described [13] and according to an approved protocol by the institutional animal care and use committee. Under methohexital sodium (50 mg kg^-1^, i.p.) anesthesia, a small surgical incision was made in the center of the scrotum. Each testicle was exposed through the surgical orifice. The ductus deferens and main arteries and veins were isolated and ligated. Subsequently, the duct and blood vessels were severed allowing the testicle and epididymis to be removed. The incision was then closed, sutured and swabbed with povidone-iodine solution. The sham operation involved the exposure of the testes without isolation. The post-operative procedure was implemented as previously described. Finally, the rats were housed in separate cages and allowed free access to food and water.

### Radioimmunoassays

The commercially available radioimmunoassay "Coat-A-Count Total Testosterone", "Coat-A-Count Direct Androstenedione" and "Double Antibody Estradiol" kits were used for the analysis of serum testosterone, androstenedione, and 17β-estradiol, respectively, and were purchased from Diagnostic Products Corporation (Los Angeles, CA).

### Protocols and experimental groups

#### Effect of testosterone replacement on baroreceptor reflex control of heart rate

Two groups of conscious unrestrained rats (orchiectomized 3 weeks earlier at 250–275 g; orchiectomized/testosterone: n = 21 and orchiectomized/vehicle: n = 14), weighing 325–350 g, were used in this experiment to investigate whether (i) testosterone replacement restores the baroreflex heart rate response, and (ii) 17β-estradiol contributes to testosterone restoration of baroreflex sensitivity. Silastic implants (inner diameter, 2.6 mm; length, 25 mm; outer diameter, 3.1 mm; Konigsberg Instruments, Inc, Pasadena, CA) containing crystallized testosterone (or empty implants for controls) were subcutaneously implanted (for 1 week) within the orchiectomized rats according to the method used in previous studies [14]. The post-operative procedure was implemented as previously described. On the day of the experiment, the rats were allowed to acclimatize to laboratory conditions for at least 2 h prior to experimentation. Subsequently, the arterial catheter was connected to a pressure transducer for measurement of blood pressure and heart rate. Blood samples (0.6 ml) were collected, from the femoral artery, for measurements of serum testosterone and 17β-estradiol levels. A period of 30 min was then allowed for further stabilization of blood pressure and heart rate. Baroreflex curves were constructed in all rats by the i.v. bolus injection of randomized doses of phenylephrine (1–16 μg kg^-1^) at 5-min intervals. Phenylephrine was dissolved in saline and administered in varying volumes of a stock concentration (36 μg ml^-1^) of phenylephrine to achieve the desired doses. Each experiment lasted approximately 1 h. The peak changes in mean arterial pressure and heart rate, obtained following phenylephrine injections, were used for the construction of the baroreflex curves.

#### Effect of long-term castration on baroreceptor reflex control of heart rate

Two groups of rats (orchiectomized n = 12, or sham-operated n = 5, 6 weeks earlier at 175–200 g), weighing 325–350 g on the day of the experiment, were used to investigate whether a reflex adrenocortical increase in testosterone or androstenedione, subsequent to long-term castration, results in a proportionate increase in baroreflex sensitivity. Blood samples (0.6 ml) were collected, from the femoral artery, prior to each experiment for the analysis of serum testosterone and androstenedione levels. Baroreflex-mediated bradycardia was assessed as previously described.

### Drugs

Phenylephrine hydrochloride (Sigma Chemical Co., St. Louis, MO), crystalline testosterone (Sigma Chemical Co., St. Louis, MO), methohexital sodium (Eli Lilly and Company, Indianapolis, IN), Buprenex (buprenorphine hydrochloride; Rickitt & Colman, Richmond, VA), povidone-iodine solution (Norton Co., Rockford, IL), and Durapen (penicillin G benzathine and penicillin G procaine; Vedco, Overland Park, KS) were purchased from commercial vendors.

### Statistical analysis

Values are expressed as means ± SEM. The relationship between increases in mean arterial pressure **(Mean Arterial Pressure = diastolic pressure + one-third {systolic - diastolic pressures}) **and associated decreases in heart rate was assessed by regression analysis for individual animals as described in our previous studies [4]. The regression coefficient (slope of the regression line) expressed as beats min^-1 ^mmHg^-1 ^was taken as an index of baroreflex-mediated bradycardia [4]. Analysis of variance (ANOVA) followed by Fisher's Least Significant Difference post hoc analysis was used for multiple comparisons. The Student's t-test was used in the analysis of unpaired data, with the level of significance set at P < 0.05. In accordance with reported criteria, control rats possessing (i) baroreflex sensitivity values with significant (P < 0.05) correlation coefficients greater than or equal to 0.8 [8], and (ii) serum testosterone levels greater than or equal to 60 ng/dl [15] were included in the data analysis.

## Authors' contributions

ARA conceived of the study and, along with GRW, participated in the design and coordination of the study as well as the drafting of the manuscript. GRW carried out all of the experiments and the statistical analyses. Both authors read and approved the final manuscript.
